# Biomechanical evaluation of different oblique lumbar interbody fusion constructs: a finite element analysis

**DOI:** 10.1186/s12891-024-07204-8

**Published:** 2024-01-27

**Authors:** Zhengquan Xu, Qingcong Zheng, Liqun Zhang, Rongsheng Chen, Zhechen Li, Weihong Xu

**Affiliations:** Department of Spinal Surgery, The First Affiliated Hospital of Fujian Medical University, Fuzhou, 350004 China

**Keywords:** Finite element analysis, Oblique lateral interbody fusion, Biomechanical evaluation, Adjacent segment degeneration

## Abstract

**Background:**

Finite element analysis (FEA) was performed to investigate the biomechanical differences between different adjunct fixation methods for oblique lumbar interbody fusion (OLIF) and to further analyze its effect on adjacent segmental degeneration.

**Methods:**

We built a single-segment (Si-segment) finite element model (FEM) for L4-5 and a double-segment (Do-segment) FEM for L3-5. Each complete FEM was supplemented and modified, and both developed two surgical models of OLIF with assisted internal fixation. They were OLIF with posterior bilateral percutaneous pedicle screw (TINA system) fixation (OLIF + BPS) and OLIF with lateral plate system (OLIF + LPS). The range of motion (ROM) and displacement of the vertebral body, cage stress, adjacent segment disc stress, and spinal ligament tension were recorded for the four models during flexion/extension, right/left bending, and right/left rotation by applying follower load.

**Results:**

For the BPS and LPS systems in the six postures of flexion, extension, right/left bending, and right/left rotation, the ROM of L4 in the Si-segment FEM were 0.32°/1.83°, 0.33°/1.34°, 0.23°/0.47°, 0.24°/0.45°, 0.33°/0.79°, and 0.34°/0.62°; the ROM of L4 in the Do-segment FEM were 0.39°/2.00°, 0.37°/1.38°, 0.23°/0.47°, 0.21°/0.44°, 0.33°/0.57°, and 0.31°/0.62°, and the ROM of L3 in the Do-segment FEM were 6.03°/7.31°, 2.52°/3.50°, 4.21°/4.38°, 4.21°/4.42°, 2.09°/2.32°, and 2.07°/2.43°. BPS system had less vertebral displacement, less cage maximum stress, and less spinal ligament tension in Si/Do-segment FEM relative to the LPS system. BPS system had a smaller upper adjacent vertebral ROM, greater intervertebral disc stress in terms of left and right bending as well as left and right rotation compared to the LPS system in the L3-4 of the Do-segment FEM. There was little biomechanical difference between the same fixation system in the Si/Do-segment FEM.

**Conclusions:**

Our finite element analysis showed that compared to OLIF + LPS, OLIF + BPS (TINA) is more effective in reducing interbody stress and spinal ligament tension, and it better maintains the stability of the target segment and provides a better fusion environment to resist cage subsidence. However, OLIF + BPS (TINA) may be more likely to cause adjacent segment degeneration than OLIF + LPS.

## Introduction

Twenty years ago, lumbar interbody fusion (LIF) was known as the most advanced technique in spinal surgery [1, 2], and developments in materials and techniques have enabled spinal fusion to progress and be versatile and elective [3]. Mayeret al*.* first performed anterior lumbar surgery via a retroperitoneal approach in 1997 [4], and Silvestreet al*.* developed and refined the technique and named it oblique lumbar interbody fusion in 2012. The early developments of OLIF were the use of a "sliding window" with a 4 cm incision and the expansion of the surgical field by floating ribs [5]. The main differences between OLIF and traditional LIF are the surgical approach, the effect of deformity correction, the degree of decompression, and complications [6]. OLIF is accessed through the anatomical gap between the psoas major muscle and the aorta/inferior vena cava (IVC) into the L2-5 intervertebral disc space to avoid damage to the lamina and paravertebral muscles [7, 8]. OLIF uses an oversized cage to support the intervertebral space to provide an indirect decompression effect and maintain the structural integrity of the posterior column, which is excellent for sagittal and coronal deformity correction [6, 9]. OLIF has the advantage of high fusion rates and few complications, with the most common complication being cage subsidence (4.4%) [10]. In addition, lumbar instability and cage subsidence frequently occur during bone remodeling following lumbar interbody fusion, and the incidence of these complications can be decreased by employing an adjunctive internal fixation system [11]. Magerlet al*.* first reported percutaneous pedicle screw placement in 1982 [12]. After 20 years of research and development, the fourth generation of the PPS system has been successfully implemented in clinical practice [13]. The TINA minimally invasive posterior spinal internal fixation system is a new PPS system that has been applied in the OLIF + BPS technique, with fewer correlation studies reported. In addition, lateral plate-screw systems (e.g., cage with lateral plate and two lateral screws, lateral plate system) can increase the lumbar spine's stability in all directions of motion after OLIF surgery [14, 15]. Some studies have reported that LPS can be used as an alternative to BPS systems in OLIF [16]. We were interested in the biomechanical differences between the OLIF + BPS and OLIF + LPS surgical approaches in terms of lumbar spine stability and degeneration of adjacent segments.

FEA was first applied to biomechanics in 1972 by Brekelmanset al. [17]. Finite element modelling and biomechanical testing can not only provide insights into understanding the complex structure of the spine, but can also make an important contribution to the design, function and application of spinal instrumentation in its preliminary stages [18]. The use of FEA in lumbar spine biomechanics is closely related to the trend of new technologies and concepts in the lumbar spine [19–22]. We established L4-5 FEM and L3-5 FEM to simultaneously compare the effects of OLIF + BPS (TINA) and OLIF + LPS on the stability of the target lumbar segments, the forces in the intervertebral space, the tensions in the ligaments, and the stresses on the upper neighbouring segments. Our study provides reliable information from a biomechanical point of view for the comparison of the effectiveness of OLIF combined with two fixation systems, providing valuable insights for clinicians and promoting the safe application of these surgical methods.

## Materials and methods

### Development of the lumbar spine FEM

A healthy adult male volunteer (age 30 years, height 172 cm, weight 75 kg, no lumbar spine disease) was selected. The spine of the volunteer was scanned using a General Electrics 64-layer spiral CT machine (scanning conditions: 120 kV, 125 mA, layer thickness: 0.625mm, top-down spiral axial scanning), and the lumbar spine was extracted from L3-5. After interpolation and enlargement of the original data, a continuous image with layer thickness was produced and saved on a CD-ROM in the international standard DICOM format.

**Fig. 1:**
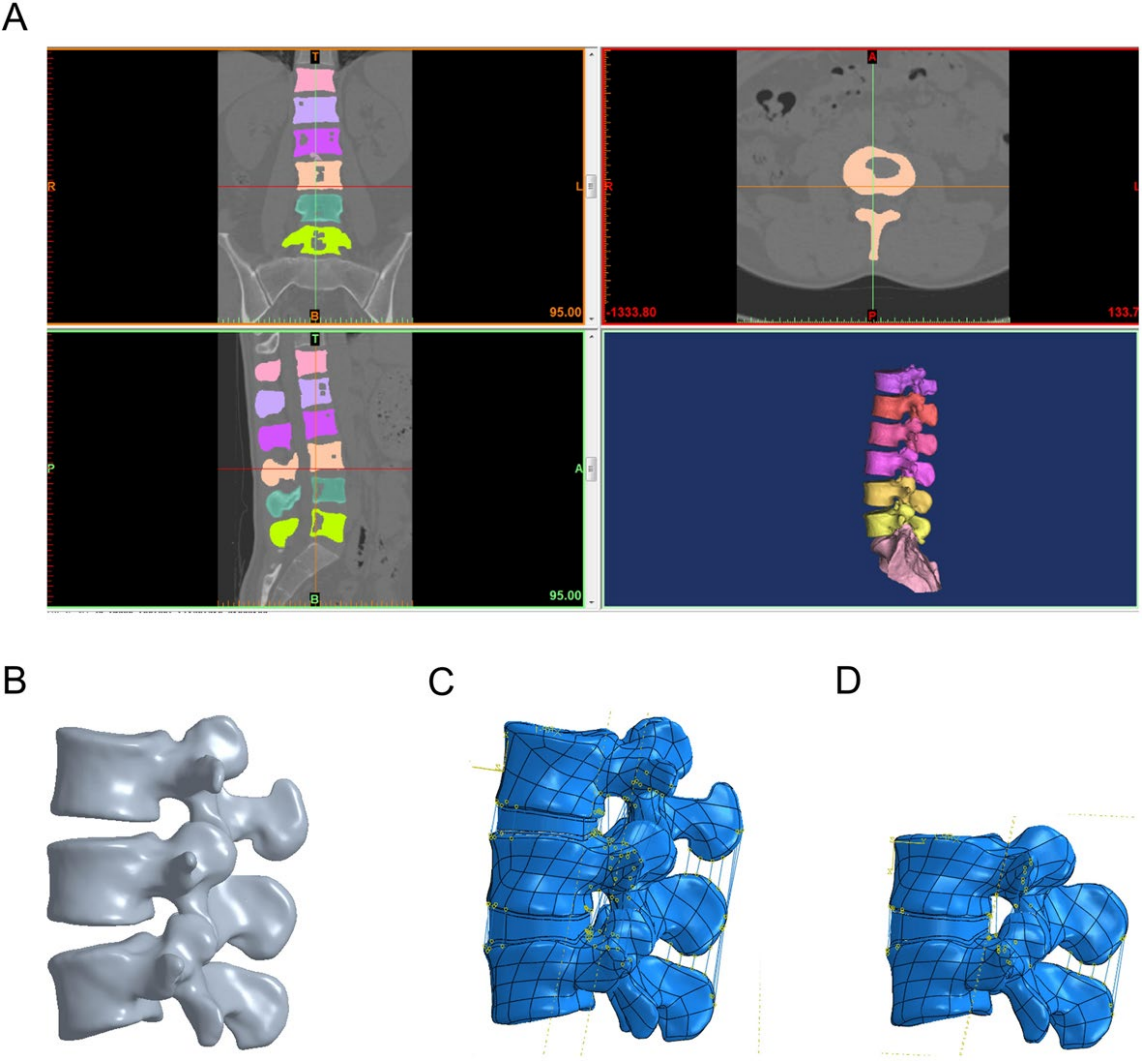
A complete 3D nonlinear finite element model of the lumbar spine. **A** Capture the skeletal structure of L3-5 lumbar spine and generate the basic 3D contour model; **B** Construct a surface model with continuity; **C** Generate a double-segment (L3-5) model; **D** Generate a single-segment (L4-5) model

The CT tomographic images of the lumbar spine in DICOM format were imported into the medical image control system Mimics 17.0 (Materialise Inc., Leuven, Belgium), and a denoising process was performed to define the optimal bone and soft tissue boundary thresholds and to remove the soft tissue images surrounding the bones. The images were selectively thresholded for segmentation according to the anatomical structure, and further operations such as region growing, photosmoothing each part of the lumbar spine, and filling the gaps so that the outer contour lines of the vertebrae were smooth and continuous were performed to capture the skeletal structure of the L3-5 lumbar spine and generate the basic 3D contour model (Fig. 1A). The constructed vertebral information was saved in STL format, and the STL format file was imported into Geomagic Studio 2012 (Geomagic Inc., NC, USA), and the model was repaired and optimized by removing lumps and indentations, smoothing the relaxed surface, and fitting the surface triangles with smooth surfaces to produce a model with a continuous surface (Fig. 1B). A series of solid models of cortical bone, cancellous bone, intervertebral discs, end plates, etc. were constructed using the computer-aided design software SolidWorks 2016 (Dassault Systèmes SolidWorks Corporation, Waltham, Massachusetts, USA). The generated solid model was imported in IGES format into the FEA software Abaqus 6.14 (Simulia, Suresnes, France) to perform model assembly, material property definition, loading and FE analysis.

Structural reconstructions of the anterior longitudinal ligament, posterior longitudinal ligament, ligamentum flavum, supraspinous ligament, interspinous ligament, and right and left intertransverse ligaments were performed in the L3-5 solid model. Endplates, posterior elements, cortical bone, and cancellous bone were classified as isotropic homogeneous elastic materials [19, 23]. The thickness of the cortical bone was 1.0 mm, the thickness of the endplate was 0.5 mm [24], the thickness of the small articular cartilage was 0.25 mm, and the small joint gap was 0.5 mm. the interaction between the joints was defined as surface contact with a coefficient of friction of zero [8, 22]. The nucleus pulposus was treated as a linearly elastic fluid element, and the fibrous annulus was represented by a mixture of matrix and collagen fibers buried in the matrix, which travel in a scissor-like fashion in the annulus and at an average ± 30° angle to the disc plane [20, 25]. Fibrillar collagen fibers and ligaments were defined as spring elements with nonlinear properties [23, 26]. The nucleus pulposus accounts for 40% of the disc volume and the annulus fibrosus for 60% [22]. An whole double-segment (L3-5) FEM of the lumbar spine was produced as a consequence, comprising the cortical bone, cancellous bone, endplates, intervertebral discs, posterior complex structures, and spinal ligaments (Fig. 1C). The Si-segment (L4-5) FEM was based on the Do-segment (L3-5) FEM with the removal of the L3 vertebral body and structures such as the disc and ligaments between L3 and L4 (Fig. 1D). We performed simultaneous comparative analyses using a Si-segment (L4-5) and a Do-segment (L3-5) FEM, not only to obtain more valid evidence by expanding the range of vertebral segments, but also to capture stresses and ROM data for adjacent segments by using a double-segment model.

### Material properties of the lumbar spine FEM

The values of modulus of elasticity and Poisson's ratio for different materials were set using data that were accepted and validated by most researchers. The cage material property is PEEK, and the relevant metal material for both OLIF's BPS system and LPS system is Ti-6Al-4V. The FEM constructed in this experimental study did not take into account the decrease in strength of vertebral cortical and cancellous bones due to osteoporosis, etc. The above material parameters were shown in Table 1.

**Table 1:** Material properties, mesh type and elements type of the lumbar spine model

Components	Young’s modulus (MPa)	Cross‐section area (mm2)	Poisson’s ratio	Element type
Cortical bone	12000	–	0.3	C3D10
Cancellous bone	100	–	0.2	C3D10
Posterior element	3500	–	0.25	C3D10
Bone endplate	500	–	0.25	C3D10
AF (substrate)	4.2	–	0.45	C3D10
AF (outer layer)	550	–	0.3	C3D10
AF (intermediate layer)	454	–	0.3	C3D10
AF (inner layer)	357	–	0.3	C3D10
Nucleus pulposus	1.0	–	0.49	C3D10
Anterior longitudinal ligament	7.8	63.7	0.3	T3D2
Posterior longitudinal ligament	10.0	20	0.3	T3D2
Ligamentum flavum	15.0	40	0.3	T3D2
Interspinous ligament	10.0	40.0	0.3	T3D2
Supraspinal ligament	8.0	30	0.3	T3D2
Intertransverse ligaments	10.0	1.8	0.3	T3D2
TINA/lateral plate	110000	–	0.3	C3D10
Cage(PEEK)	3900	–	0.4	C3D10

*Abbreviation*: *AF* Annulus fibers, *PEEK* Polyetheretherketone

### Development of the surgical lumbar spine FEM

**Fig. 2:**
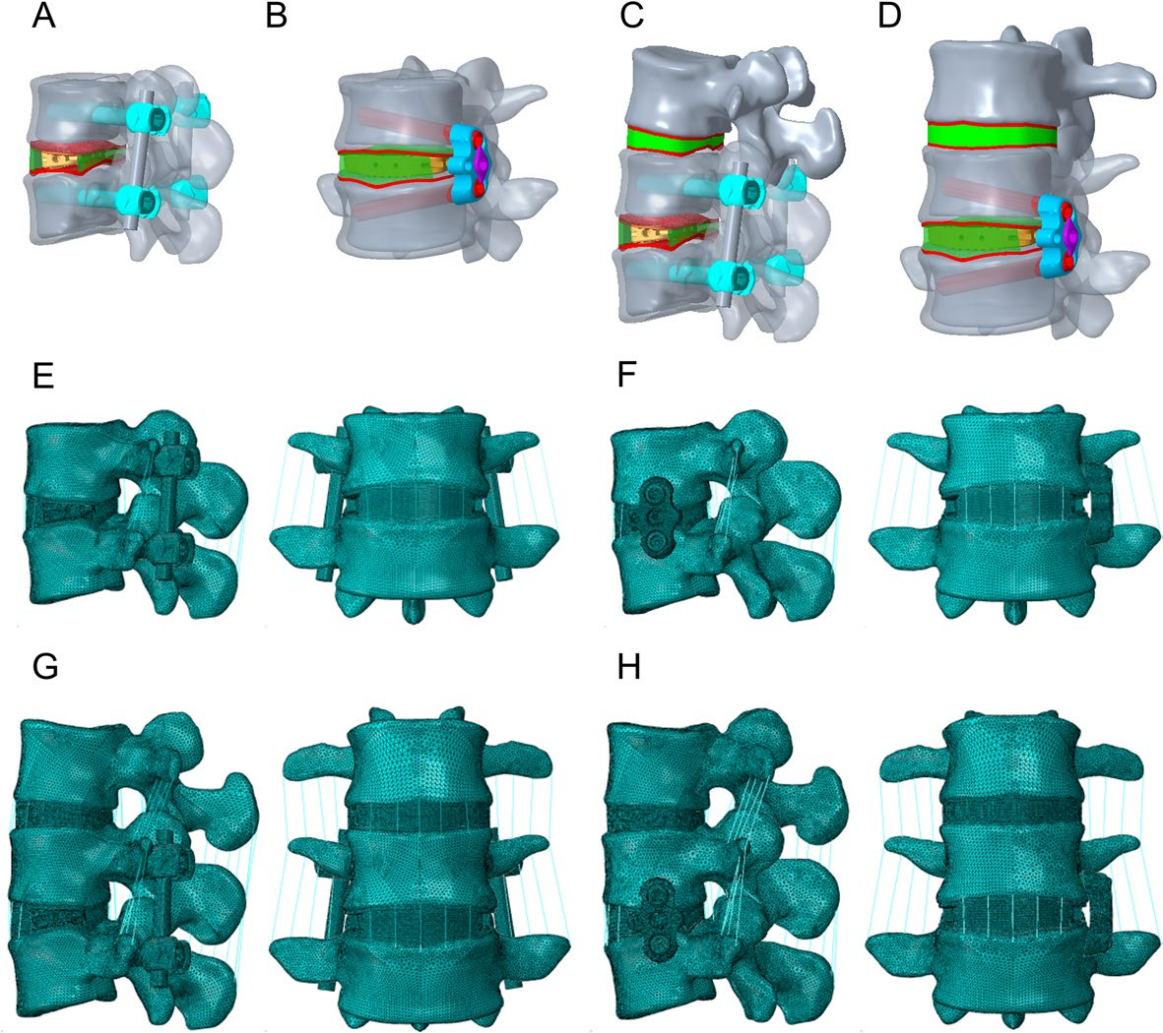
Four types of finite element models. Single-segment model: **A** OLIF combined with posterior bilateral pedicle screws (TINA system) (OLIF + BPS); **B** OLIF combined with lateral plate system (OLIF + LPS). Double segment model: **C** OLIF + BPS; **D** OLIF + LPS. Single-segment model: **E** mesh details diagram for OLIF + BPS; **F** mesh details diagram for OLIF + LPS. Double segment model: **G** Mesh details diagram for OLIF + BPS; **H** Mesh details diagram for OLIF + LPS

Two surgical models, OLIF + BPS (TINA) and OLIF + LPS, were further simulated based on the validity of the previously validated models. A portion of the disc between L4-5 was removed and a reasonable-sized cage was implanted. The cage was modelled using Oracle cage (DePuy Synthes). It is made of polyetheretherketone with parameters of 8° lordosis, 40 mm long, 22 mm wide, 11 mm high in front and 8 mm high in back. We placed the cage in the FEM through 15° to the coronal plane based on the CT images. The BPS system comprised two rods and four pedicle screws. The pedicle screws were set to enter from the position of the pedicle plate and, via a direction parallel to the upper surface of the vertebral body, pass through the centre of the cross-section of the pedicle into the vicinity of the anterior cortical bone of the vertebral body, and were not penetrable. The pedicle screws were 6.5 mm in diameter and 50 mm in length, and the rods were 5.5 mm in diameter and 52 mm in length. The metal spinal plates used in the LPS system have nail holes at both ends, and the angle of the centre axis of the two nail holes is designed to be 20°, so that the two vertebrae can be stably braced, and the inner side of the plate is curved so that it can fit well with the vertebral body surfaces to ensure the stability of the vertebral body after being subjected to stresses. The metal spinal plates were placed on the left side of the vertebral body, and screws were driven along the location of the screw holes, setting the screws to pass through the cancellous bone and into the right side of the vertebral body near the cortical bone. The two screws were 6.5 mm in diameter and 40 mm in length (Fig. 2A-D). Our study establishes a Si-segment (L4-5) and a Do-segment (L3-5) FEM with precise geometrical profiles, comprehensive biomechanical properties under the principle of meshing, and Table 2 and Fig. 2E-H show the mesh details. The lumbar mobility, interbody stresses, spinal ligament tensions, and upper adjacent segmental stresses were compared and contrasted between the four FEMs in six postures: flexion, extension, right/left bending, and right/left rotation, and whether there were differences between the same surgical approach in the Si-segment model and the Do-segment model.

**Table 2:** Mesh details of components in the lumbar spine model

Components	Element type	Number of elements	Number of nodes
L3 cortical bone	C3D10	236059	51933
L4 cortical bone	C3D10	249132	54809
L5 cortical bone	C3D10	266887	58715
L3 cancellous bone	C3D10	99065	21794
L4 cancellous bone	C3D10	100701	22154
L5 cancellous bone	C3D10	107005	23541
BPS	C3D10	390945	86008
LPS	C3D10	253478	55765
Intervertebral disc	C3D10	276612	60855
Cage	C3D10	271155	59654
Endplate	C3D10	213646	47002
Ligament	T3D2	90	180

### Boundary and loading conditions of the lumbar spine FEM

The meshing of the FEM was completed in the meshing module of ABAQUS 6.14, followed by mesh checking. The mesh sensitivity analysis software used in this study evaluates the mesh quality with two metrics, analysis errors and analysis warnings, and the result was that the analysis errors = 0 and the analysis warnings < 0.2%, which indicates that the mesh quality was satisfactory. The boundary conditions were the degrees of freedom constraining the motion of all nodes of the lower surface of the L5 vertebrae in three directions. The physiological loading condition was to apply an axial load of 400 N to the upper surface of the uppermost vertebra of each FEM to simulate the vertical load of physiological compression (upright state), and a moment condition of 10 N.m to simulate the lumbar spine in six postures: flexion, extension, right/left bending, and right/left rotation. The loads applied in this study have been shown to be sufficient to produce physiological range of motion without causing spinal instability [27]. Our study was measured using the biplane stereo method created by Panjabi et al. [28].

### Model validation

**Fig. 3:**
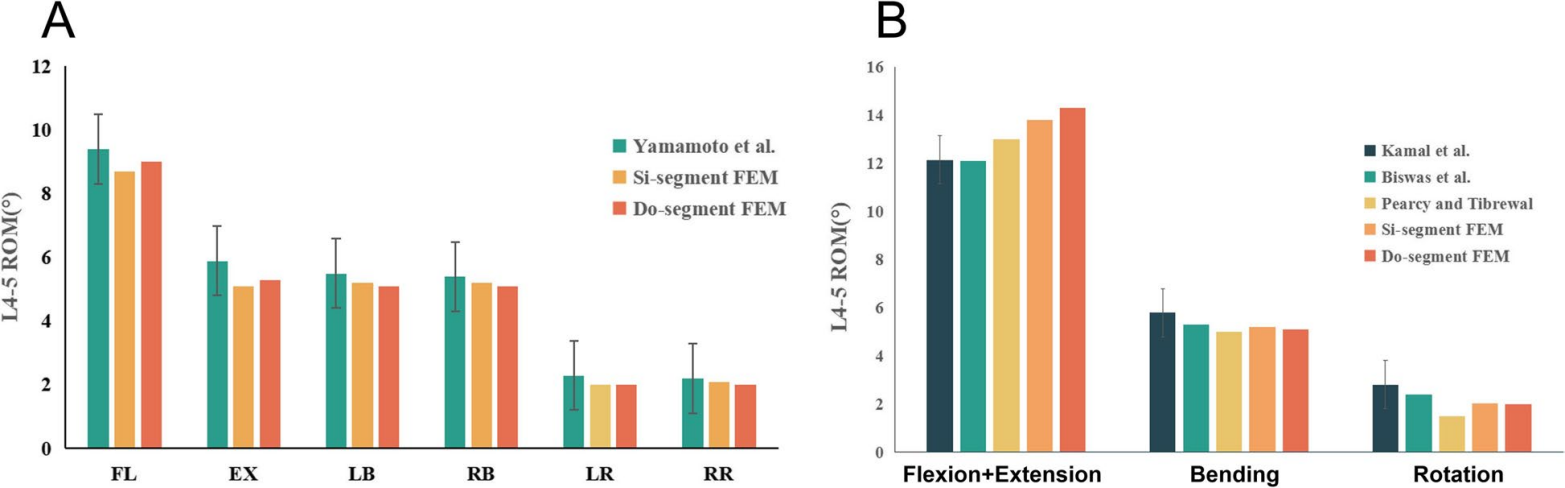
Comparison of the predicted results of the constructed finite element model with the investigation of Yamamoto et al.

The Si-segment (L4-5) FEM has 657,152 total elements and 149,609 nodes and the Do-segment (L3-5) FEM has 997,650 total elements and 233,898 nodes. We compared the ROM measurements of these two FEMs with the results of Yamamoto et al. [29] and presented them in Table 3 and Fig. 3A, and found that the three results were basically the same. In addition, further comparing the present study model with those of Kamal et al. [30], Biswas et al. [31] and Pearcy and Tibrewal [32], our results likewise fall within the range of variation (Table 4 and Fig. 3B). The validity and robustness of the model was demonstrated and it can be applied to the next step of biomechanical analyses of the lumbar spine.

**Table 3:** Comparison of the ROM of the complete finite element model in the present study with the data of Yamamoto et al. (degree)

Model	Flexion	Extension	Left bending	Right bending	Left rotation	Right rotation
Yamamoto I	9.4	5.9	5.5	5.4	2.3	2.2
Si-segment FEM	8.7	5.1	5.2	5.2	2.0	2.1
Do-segment FEM	9.0	5.3	5.1	5.1	2.0	2.0

**Table 4:** Comparison of the ROM of the complete finite element model in the present study with data from other studies (degree)

Model	Flexion + Extension	Bending	Rotation
Kamal et al	12.15	5.80	2.82
Biswas et al	12.1	5.3	2.4
Pearcy and Tibrewal	13	5	1.5
Si-segment FEM	13.8	5.2	2.05
Do-segment FEM	14.3	5.1	2.0

## Results

### Displacement and ROM

**Fig. 4:**
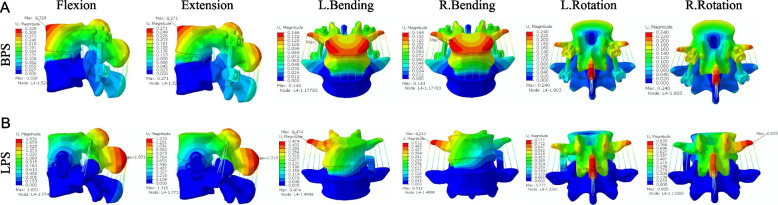
Simulation plots of displacement and ROM of L4 in the single-segment (L4-5) model. **A** OLIF + BPS; **B** OLIF + LPS

**Fig. 5:**
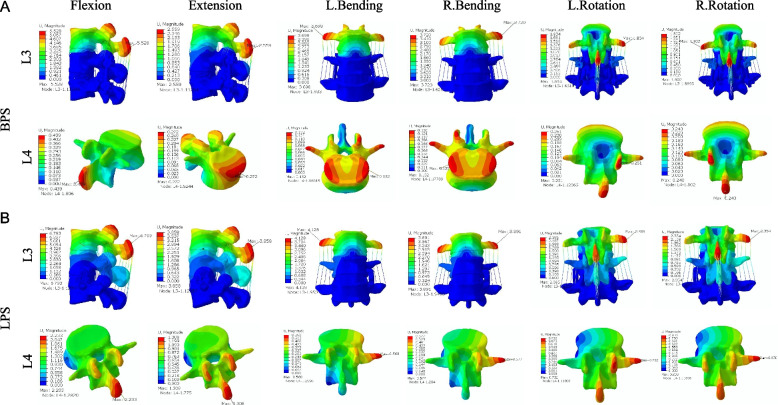
Simulation plots of displacement and ROM of L3 and L4 in the double-segment (L3-5) model. **A** OLIF + BPS; **B** OLIF + LPS

**Fig. 6:**
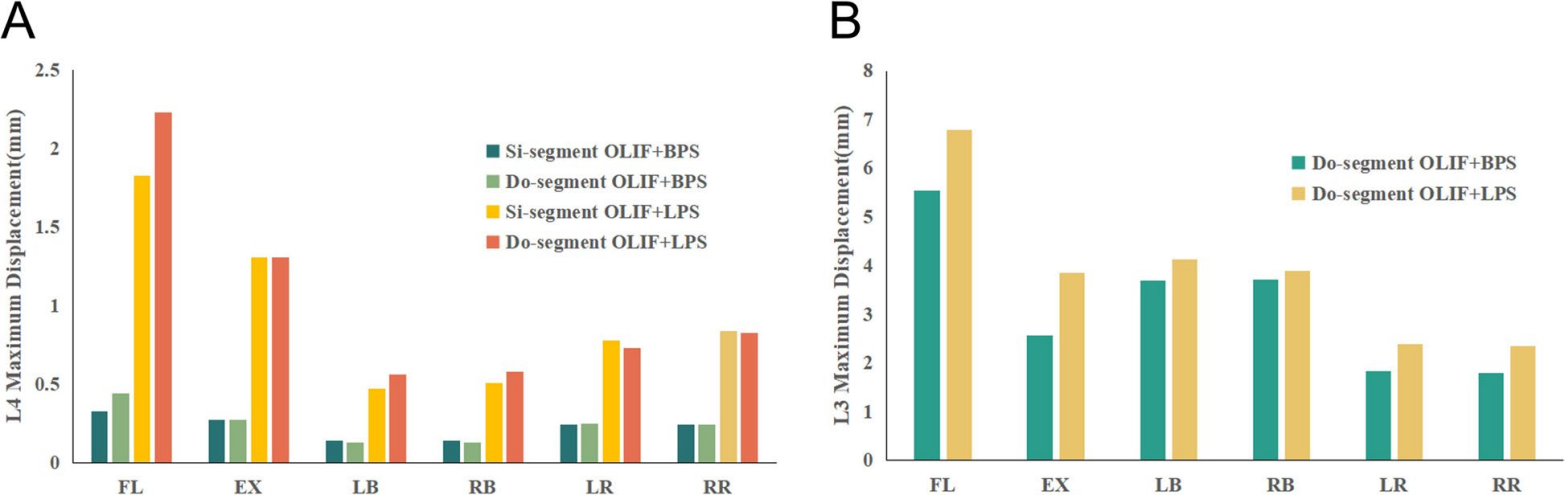
**A** Displacement of the vertebrae of the target segment in the four models. The horizontal coordinate is the six common directions of motion and the vertical coordinate is the maximum displacement (mm) of L4. **B** Displacement of vertebrae in adjacent segments in the 2 models. The horizontal coordinate is the six common directions of motion and the vertical coordinate is the maximum displacement (mm) of L3

**Fig. 7:**
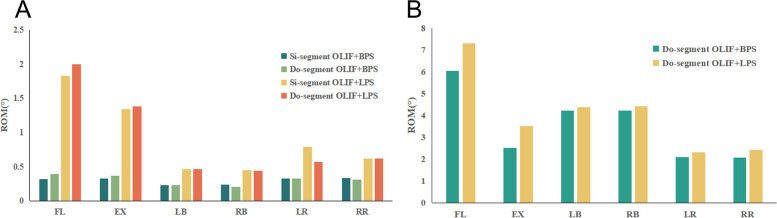
**A** ROM of the vertebrae of the target segment in the four models. The horizontal coordinate is the six common directions of motion and the vertical coordinate is the ROM (°) of L4. **B** ROM of vertebrae of adjacent segments in the 2 models. The horizontal coordinate is the six common directions of motion and the vertical coordinate is the ROM (°) of L3

To compare the difference in stability of the target segment between the two surgical approaches, we measured the displacement and ROM of the vertebral body in each model. Figure 4 shows the simulation plots of OLIF + BPS and OLIF + LPS in Si-segment (L4-5) FEM for L4 under six postures. Figure 5 shows the simulation plots of OLIF + BPS and OLIF + LPS in the Do-segment (L3-L5) FEM for L3 and L4 under six postures. Figure 6A shows the L4 displacements of the two groups under six postures in Si/Do-segment FEMs, while Fig. 6B shows the L3 displacements of the two groups in Do-segment FEMs. Figure 7A shows the ROM of L4 for two groups in the case of Si/Do-segment FEMs, while Fig. 7B represents the ROM of L3 for two groups in Do-segment FEMs. Tables 5 and 6 show the displacement and ROM of these 4 models. In the L4 of the Si-segment FEM, the displacements of OLIF + BPS and OLIF + LPS were 0.33/1.83, 0.27/1.31, 0.14/0.47, 0.14/0.51, 0.24/0.78, and 0.24/0.84 mm in six postures of flexion, extension, right/left bending, and right/left rotation, respectively. And the ROMs were 0.32°/1.83°, 0.33°/1.34°, 0.23°/0.47°, 0.24°/0.45°, 0.33°/0.79°, and 0.34°/0.62°. In the L4 of Do-segment FEM, the displacements corresponding to OLIF + BPS and OLIF + LPS were 0.44/2.23, 0.27/1.31, 0.13/0.56, 0.13/0.58, 0.25/0.73, and 0.24/0.83 mm, and the ROMs were 0.39°/2.00°, 0.37°/1.38°, 0.23°/0.47°, 0.21°/0.44°, 0.33°/0.57°, and 0.31°/0.62°. While in the L3, OLIF + BPS and OLIF + LPS correspond to displacements of the displacements were 5.53/6.79, 2.56/3.86, 3.70/4.13, 3.72/3.89, 1.83/2.39, and 1.80/2.35mm, and the ROMs were 6.03°/7.31°, 2.52°/3.50°, 4.21°/4.38°, 4.21°/4.42°, 2.09°/2.32°, and 2.07°/2.43°. We can observe that the displacement and mobility of L4 in the OLIF + BPS group's under six postures were smaller than those in the OLIF + LPS group in both Si-segment and Do-segment FEMs. In the Do-segment FEM, the displacement and mobility of L3 in the OLIF + BPS group at six postures were also smaller than those in the OLIF + LPS group. And the biomechanical differences in Si/Do-segment FEMs were not significant for the same internal fixation procedure.

**Table 5:** Displacement of vertebrae in the four models (mm)

Model	Vertebrae	Flexion	Extension	Left bending	Right bending	Left rotation	Right rotation
**Si-segment**							
OLIF + BPS	L4	0.33	0.27	0.14	0.14	0.24	0.24
OLIF + LPS	L4	1.83	1.31	0.47	0.51	0.78	0.84
**Do-segment**							
OLIF + BPS	L3	5.53	2.56	3.70	3.72	1.83	1.80
	L4	0.44	0.27	0.13	0.13	0.25	0.24
OLIF + LPS	L3	6.79	3.86	4.13	3.89	2.39	2.35
	L4	2.23	1.31	0.56	0.58	0.73	0.83

**Table 6:** ROM of vertebrae in the four models (degree)

Model	Vertebrae	Flexion	Extension	Left bending	Right bending	Left rotation	Right rotation
**Si-segment**							
OLIF + BPS	L4	0.32	0.33	0.23	0.24	0.33	0.34
OLIF + LPS	L4	1.83	1.34	0.47	0.45	0.79	0.62
**Do-segment**							
OLIF + BPS	L3	6.03	2.52	4.21	4.21	2.09	2.07
	L4	0.39	0.37	0.23	0.21	0.33	0.31
OLIF + LPS	L3	7.31	3.50	4.38	4.42	2.32	2.43
	L4	2.00	1.38	0.47	0.44	0.57	0.62

### Cage stress

**Fig. 8:**
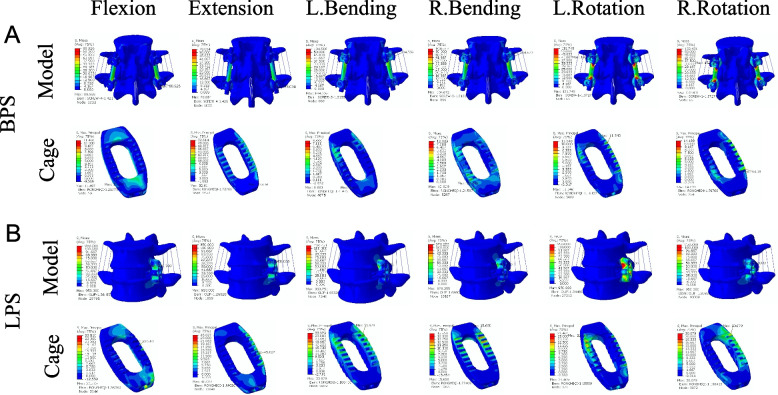
Distribution of Cage stress in two single-segment (L4-5) models. **A** Motion simulation and Cage stress distribution in OLIF + BPS. **B** Motion simulation and Cage stress distribution in OLIF + LPS

**Fig. 9:**
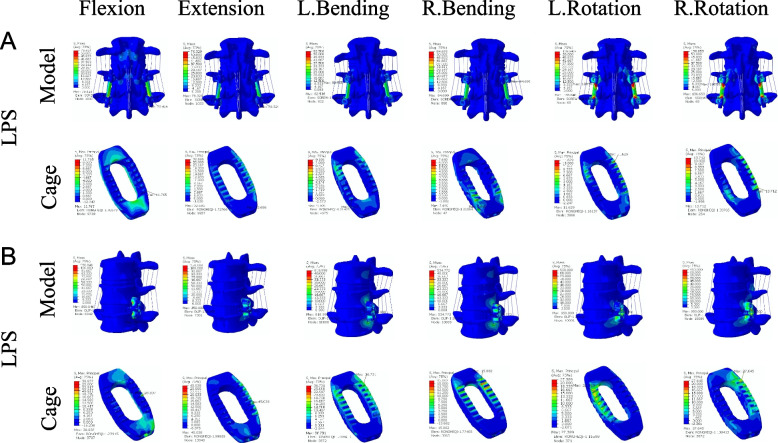
Distribution of Cage stress in two double-segment (L3-5) models. **A** Motion simulation and Cage stress distribution in OLIF + BPS. **B** Motion simulation and Cage stress distribution in OLIF + LPS

**Fig. 10:**
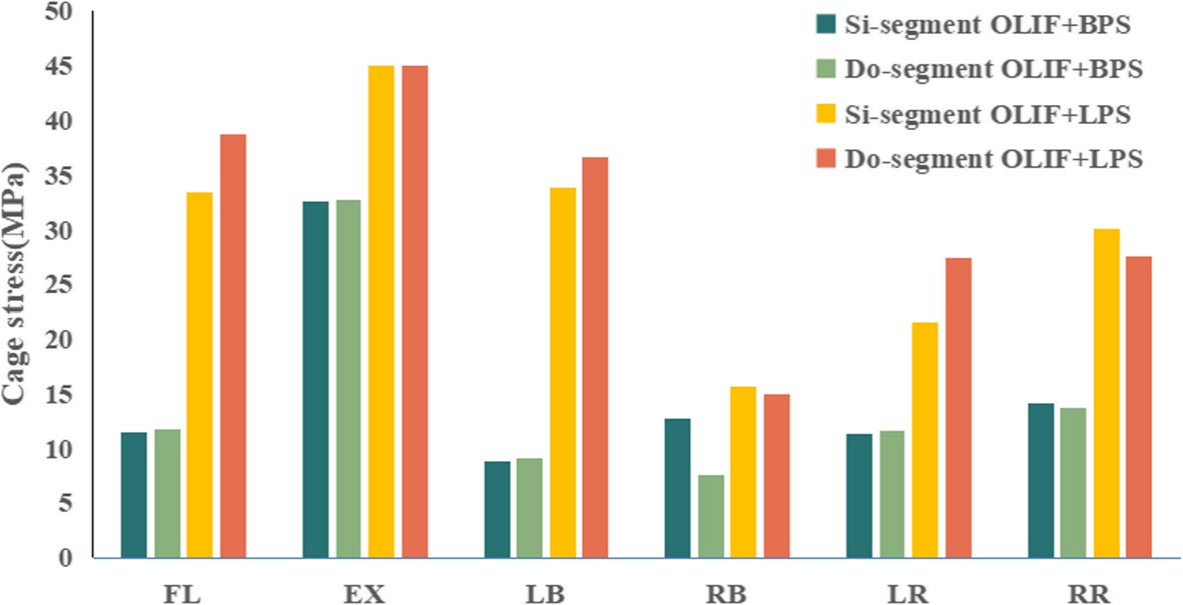
Stresses of Cage in the four models. The horizontal coordinate is the six common directions of motion and the vertical coordinate is the Cage stress values (MPa) in L4-5

To further evaluate the factors influencing lumbar fusion after OLIF surgery, we explored the stresses to which the implanted cage was subjected. Figure 8 shows the cage stress distribution clouds for OLIF + BPS and OLIF + LPS in the Si-segment (L4-5) FEM, and Fig. 9 shows the cage stress distribution clouds for the two groups in the Do-segment (L3-5) FEM at the L4-5 intervertebral space. The von mises stress distribution on cage was randomly selected and displayed by contour plots. Table 7 and Fig. 10 show the maximum stress in the cage for the two groups in the Si/Do-segment FEMs. In the Si-segment FEMs, the maximum stress values for the cage with OLIF + BPS and OLIF + LPS in flexion, extension, right/left bending, and right/left rotation were 11.5/33.5, 32.6/45.0, 8.9/33.9, 12.8/15.7, 11.3/21.5, and 14.2/30.1 MPa. In the Do-segment FEM, the maximum stress values corresponding to the two groups of cage were 11.8/38.8, 32.7/45.0, 9.1/36.7, 7.6/15.0, 11.6/27.4, and 13.7/27.6 MPa. The results showed that the maximum stresses in the cage of the OLIF + BPS group were smaller than those of the OLIF + LPS group in six postures in both the Si-segment and Do-segment models.

**Table 7:** Maximum stress values for cage in the four models (MPa)

Model	Flexion	Extension	Left bending	Right bending	Left rotation	Right rotation
**Si-segment**						
OLIF + BPS	11.5	32.6	8.9	12.8	11.3	14.2
OLIF + LPS	33.5	45.0	33.9	15.7	21.5	30.1
**Do-segment**						
OLIF + BPS	11.8	32.7	9.1	7.6	11.6	13.7
OLIF + LPS	38.8	45.0	36.7	15.0	27.4	27.6

### Intervertebral disc stress

**Fig. 11:**
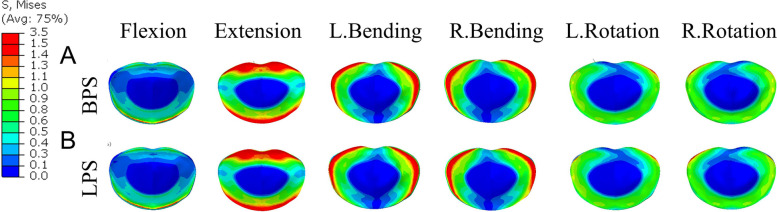
Von Mises stress distribution of the L3-4 disc in the double-segment (L3-5) model. **A** Simulation of cage stress distribution in OLIF + BPS. **B** Simulation of cage stress distribution in OLIF + LPS

**Fig.12:**
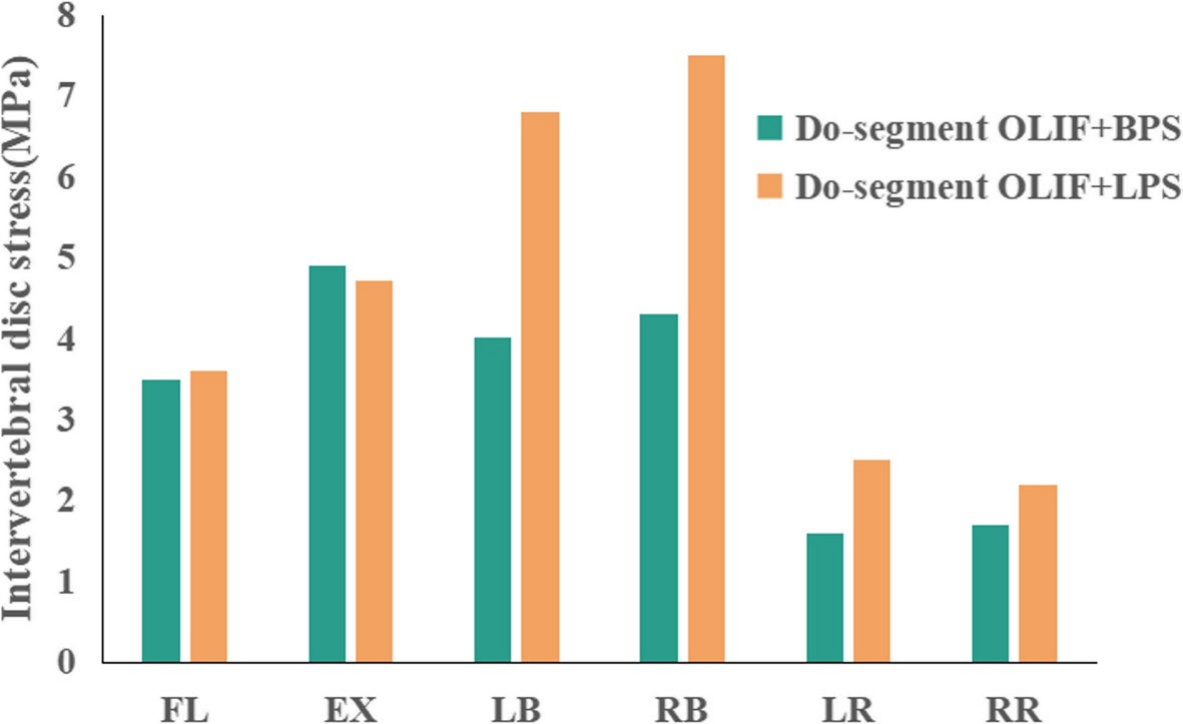
Stresses in the discs of adjacent segments in the two models. The horizontal coordinate is the 6 common directions of motion, and the vertical coordinate is the stress value (MPa) of the L3-4 intervertebral disc

To further compare the effects of different fixation methods of OLIF on degenerative changes in adjacent segments, we explored differences in the stress profile of the intervertebral disc. Fig. 11 shows the stress clouds of the L3-4 disc in the Do-segment (L3-5) FEM in the case of six postures for OLIF + BPS and OLIF + LPS. and the von mises stress distribution on the disc is shown by contour plots. In the Do-segment FEM, the maximum stress values corresponding to the intervertebral discs in the upper neighboring segments of OLIF + BPS and OLIF + LPS were 3.6/3.5, 4.7/4.9, 6.8/4.0, 7.5/4.3, 2.5/1.6, and 2.2/1.7 MPa (Table 8 and Fig. 12). From their stress distribution on the L3-4 discs of the upper adjacent segments, it can be seen that there was no significant difference in anterior flexion and posterior extension, while the OLIF + LPS group was smaller than the OLIF + BPS group in left bending and right bending as well as in left and right rotation.

**Table 8:** Maximum stresses in the L3-4 disc in the two models (MPa)

Model	Flexion	Extension	Left bending	Right bending	Left rotation	Right rotation
**Do-segment**						
OLIF + BPS	3.6	4.7	6.8	7.5	2.5	2.2
OLIF + LPS	3.5	4.9	4.0	4.3	1.6	1.7

### Spinal ligament tension

**Fig. 13:**
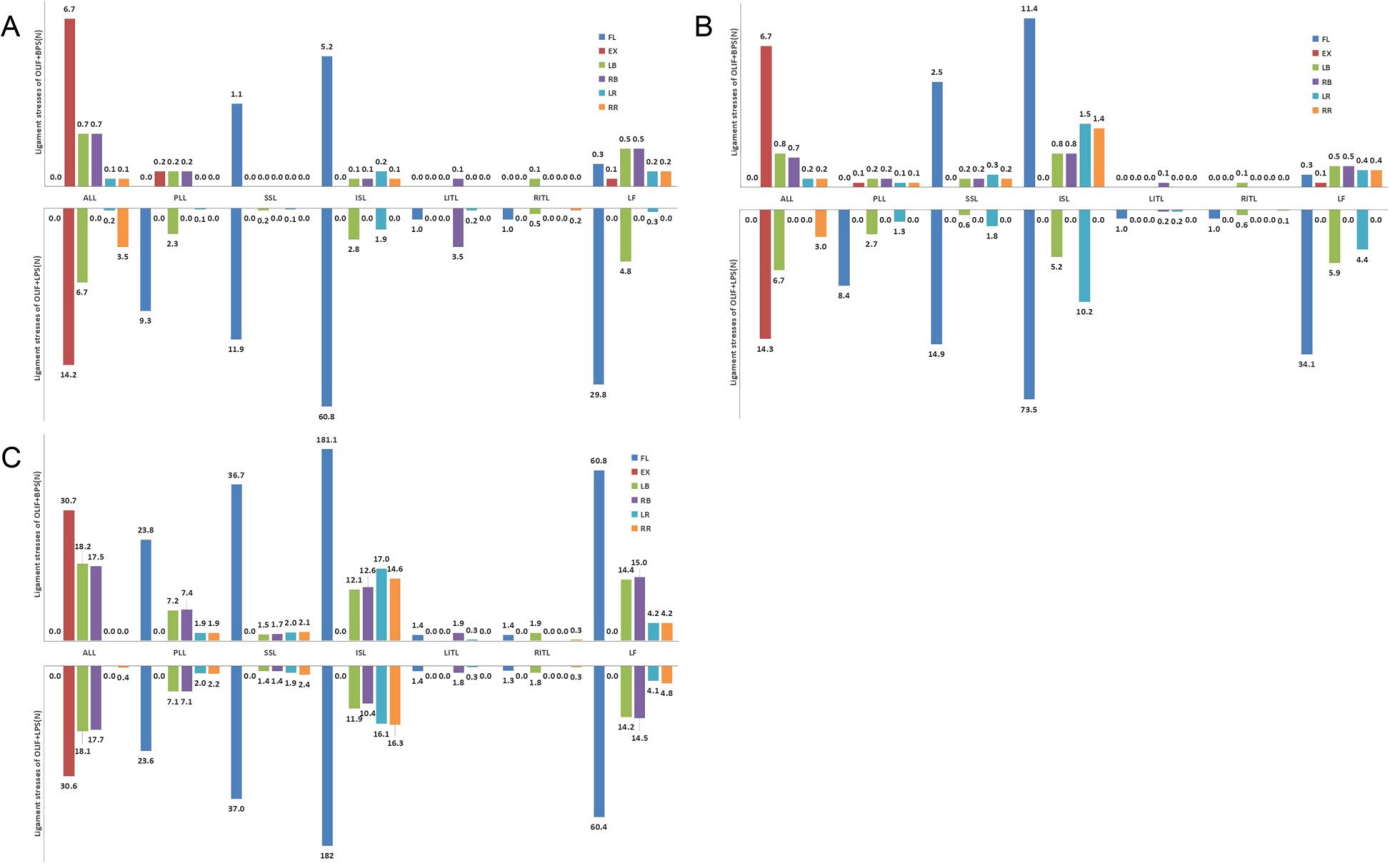
Spinal ligament tensions in the 4 models. **A** Tensions in the L4-5 spinal ligaments in the single-segment (L4-5) model; **B** Tensions in the L4-5 spinal ligaments in the double-segment (L3-5) model; **C** Tensions in the L3-4 spinal ligaments in the double-segment (L3-5) model. The horizontal coordinate is the 7 spinal ligaments, and the positive axis of the vertical coordinate is OLIF + BPS and the negative axis is OLIF + LPS

In addition to the intervertebral disc, we further investigated the differences in tensions on the spinal ligaments of the target and adjacent segments by different fixation methods in OLIF. From the data in Fig. 13 as well as Table 9, it was clear that the OLIF + BPS group had less overall spinal ligament tension than the OLIF + LPS group in all six postures, both in Si-segment and Do-segment FEMs. And in Do-segment FEM, little difference was found between OLIF + BPS and OLIF + LPS groups for the spinal ligaments of L3-4.

**Table 9:** Tension values for each spinal ligament in the four models (N)

Model	Location	Ligament	Flexion	Extension	Left bending	Right bending	Left rotation	Right rotation
**OLIF + BPS**								
Si-segment	L4-5	ALL	0.0	6.7	0.7	0.7	0.1	0.1
		PLL	0.0	0.2	0.2	0.2	0.0	0.0
		SSL	1.1	0.0	0.0	0.0	0.0	0.0
		ISL	5.2	0.0	0.1	0.1	0.2	0.1
		LITL	0.0	0.0	0.0	0.1	0.0	0.0
		RITL	0.0	0.0	0.1	0.0	0.0	0.0
		LF	0.3	0.1	0.5	0.5	0.2	0.2
Do-segment	L3-4	ALL	0.0	30.7	18.2	17.5	0.0	0.0
		PLL	23.8	0.0	7.2	7.4	1.9	1.9
		SSL	36.7	0.0	1.5	1.7	2.0	2.1
		ISL	181.1	0.0	12.1	12.6	17.0	14.6
		LITL	1.4	0.0	0.0	1.9	0.3	0.0
		RITL	1.4	0.0	1.9	0.0	0.0	0.3
		LF	60.8	0.0	14.4	15.0	4.2	4.2
	L4-5	ALL	0.0	6.7	0.8	0.7	0.2	0.2
		PLL	0.0	0.1	0.2	0.2	0.1	0.1
		SSL	2.5	0.0	0.2	0.2	0.3	0.2
		ISL	11.4	0.0	0.8	0.8	1.5	1.4
		LITL	0.0	0.0	0.0	0.1	0.0	0.0
		RITL	0.0	0.0	0.1	0	0.0	0.0
		LF	0.3	0.1	0.5	0.5	0.4	0.4
**OLIF + LPS**								
Si-segment	L4-5	ALL	0.0	14.2	6.7	0.0	0.2	3.5
		PLL	9.3	0.0	2.3	0.0	0.1	0.0
		SSL	11.9	0.0	0.2	0.0	0.1	0.0
		ISL	60.8	0.0	2.8	0.0	1.9	0.0
		LITL	1.0	0.0	0.0	3.5	0.2	0.0
		RITL	1.0	0.0	0.5	0.0	0.0	0.2
		LF	29.8	0.0	4.8	0.0	0.3	0.0
Do-segment	L3-4	ALL	0.0	30.6	18.1	17.7	0.0	0.4
		PLL	23.6	0.0	7.1	7.1	2.0	2.2
		SSL	37.0	0.0	1.4	1.4	1.9	2.4
		ISL	182.0	0.0	11.9	10.4	16.1	16.3
		LITL	1.4	0.0	0.0	1.8	0.3	0.0
		RITL	1.3	0.0	1.8	0.0	0.0	0.3
		LF	60.4	0.0	14.2	14.5	4.1	4.8
	L4-5	ALL	0.0	14.3	6.7	0.0	0.0	3.0
		PLL	8.4	0.0	2.7	0.0	1.3	0.0
		SSL	14.9	0.0	0.6	0.0	1.8	0.0
		ISL	73.5	0.0	5.2	0.0	10.2	0.0
		LITL	1.0	0.0	0.0	0.2	0.2	0.0
		RITL	1.0	0.0	0.6	0.0	0.0	0.1
		LF	34.1	0.0	5.9	0.0	4.4	0.0

*Abbreviation*: *ALL* anterior longitudinal ligament, *PLL* posterior longitudinal ligament, *SSL* supraspinous ligament, *ISL* interspinous ligament, *LITL* left intertransverse ligament, *RITL* right intertransverse ligament, *LF* ligamentum flavum

## Discussion

In the treatment of degenerative disc disease, lumbar fusion is both the common clinical procedure and the gold standard [33]. LIF needs to enhance stability by reducing the mobility of the target segment, and if the target segment is not stable after lumbar spine surgery, it will lead to complications such as delayed fusion, non-fusion, and cage subsidence, causing back pain and functional impairment in patients [34, 35]. However, the fixation of the lumbar fusion will also lead to stress overload of the adjacent segments, leading to an increased risk of adjacent segment degeneration (ASD) [33, 36, 37]. Revision surgery was required in 5.0% to 15.0% of cases following ASD after lumbar spine surgery [38, 39]. Overstretching and high-stiffness fixation of the fused segment and damage to the posterior soft tissues are important causes of ASD after LIF [40, 41].

The OLIF procedure can increase the fusion area, decrease the fusion time, increase the vertebral height recovery rate and fusion rate, and also reduce intraoperative bleeding, shorten the operation and hospital stay, and perform early recovery compared to the LIF surgical approach [42–45]. Nevertheless, cage subsidence remains one of its important postoperative complications [46]. Age > 60 years, osteoporosis, higher cage height, excessive end plate concave angle (ECA) and cage/end plate angle mismatch are important risk factors for cage subsidence in OLIF [15, 47, 48]. Although cage implantation alone can reduce vertebral mobility, the addition of posterior internal spinal fixation can significantly improve structural stability [49]. The TINA system, a new percutaneous BPS, features a dual-core, dual-wire and corrugated ball head design with a widened 6.1mm titanium rod space, a 20mm slide-off lift length and six bone cement hole channels. The TINA system allows for better vertebral support and fixation in lumbar spine surgery, faster nail screwing, easier rod placement, enhanced pull-out resistance, and reduced risk of bone cement leakage. And the addition of lateral plate system fixation to OLIF can also improve the stability of the operated segment [14]. OLIF is effective in increasing the rate of intervertebral fusion through the use of adjunctive internal fixation; however, the degree of fusion and the incidence of ASD vary depending on the material and the type of internal implant [50].

Wanget al*.* found that surgical segments fixed with the oblique lateral locking plate system had greater ROM measurements than the BPS system by constructing a FEM of the OLIF combined with an assisted fixation system, but were slightly superior to the BPS in reducing endplate stress during lumbar spine motion [16]. Our study takes into account various factors and has the following similar as well as further findings. First, the stability of the vertebral body. In the Si-segment (L4-5) and Do-segment (L3-5) FEMs of OLIF, the stability of BPS (TINA) under flexion, extension, right/left bending, and right/left rotation was better than that of LPS. BPS is more restrictive to the target segment and can provide a better fusion environment. Second, the stability of Cage. The larger the stress between the cage and the final plate, the greater the risk of cage sinking [24]. The maximum stresses in the BPS (TINA) are less than those in the LPS at six postures, probably because the posterior BPS has a stronger fixation than the lateral LPS system and more significantly shares the stresses between the cage and the end plate. This demonstrates a lower risk of cage sinking for OLIF + BPS than for OLIF + LPS. Third, the stability of the spinal ligaments. Spinal ligaments contribute important biomechanics in maintaining spinal stability [51], and intervertebral disc degeneration is also closely related to the biomechanics of spinal ligaments [52]. Thus spinal ligaments play an important role in assessing spinal stability [53, 54]. The spinal ligament tension in the BPS (TINA) group in this study was generally less than that in the LPS group, and the BPS was more effective than the LPS in sharing tensions in the spinal ligaments of the target segment.

After LIF surgery leads to increased stiffness and more stress concentration, the stress load will be transferred to the adjacent segments causing new lumbar degeneration [55]. Therefore, we have to face the issue that while using spinal instrumentation to increase the stability of the target segment to provide a robust fusion environment, it must also be considered that it will lead to an increased risk of ASD. Ghiselliet al*.* concluded that ASD has a 5-year incidence of 16.5% and a 10-year incidence of 36.1%, ASD is a complication after LIF that cannot be ignored [56]. It has been noted that L4-5 fusion will lead to accelerated degradation of L5-S1 [57]. However, as far as we know, few studies have used FEA to observe the effects of OLIF combined with different internal fixation modalities on the biomechanics of the upper adjacent vertebrae, discs, and ligaments.

In our study, the effects of using BPS and LPS in OLIF on the upper neighboring segments also differed in the following 3 points. First, the stability of the vertebral body. The displacement and ROM of L3 and L4 of the BPS (TINA) group are smaller than those of the LPS group for the same moment conditions. Therefore, under the same conditions of displacement and mobility of the vertebrae required for daily human activities, the vertebrae in the upper adjacent segment in the BPS (TINA) group require a greater moment than in the LPS group to accomplish this, which results in greater stress on the disc in the upper segment and thus accelerates the degeneration of the adjacent segment. So the BPS group was at greater risk of ASD than LPS. Second, the stability of the intervertebral disc. In addition to the generation of abnormal motion patterns in adjacent vertebrae, increased disc stress also suggests the development of degenerative changes in adjacent segments [18]; in fact, the most common lesion in adjacent segments is disc degeneration [58]. There is no significant difference in flexion and extension for BPS(TINA) compared to LPS, while the stress increases significantly in right/left bending, and right/left rotation positions. BPS is more stressful to the discs and endplates of the upper adjacent segments and is more likely to cause disc degeneration. Third, the difference between the tensions of BPS (TINA) and LPS on the ligaments surrounding the upper adjacent segment was not significant, with the possible reason being that the disc in the upper adjacent segment took most of the stress after the transfer load.

Due to the complex structure of the lumbar spine, the establishment of the FEM and the corresponding analysis have certain limitations and can only reflect some of the motion patterns as well as biomechanical changes. In addition, the muscle factor was not added to this model and may not fully simulate the stress conditions to which the normal lumbar spine is subjected in vivo. Finally, FEM provides only a predictive profile in the postoperative period and does not reflect long-term follow-up. Nevertheless, the validity of the FEM of this experiment was verified with some predictive effect. Compared to the LPS system, the BPS (TINA) system shows better stability in the fixation of the operated segments, which not only shortens the postoperative recovery time and reduces bed-ridden complications, but also allows early rehabilitation and improves the prognosis of the patient, but also carries a higher risk of degeneration of the adjacent segments. Research in this area requires further biomechanical validation in cadavers for eventual clinical application. With the development of artificial intelligence and robotics, future research could be conducted through robotic simulations to further collect biomechanical data and validate them mechanically, which may be a promising research direction. Our findings may help spine surgeons to choose the most appropriate surgical strategy and the optimal internal fixation solution for the individual variability of the patient population in clinical practice.

## Conclusions

In conclusion, the bilateral pedicle screw (TINA) is more useful than the lateral plate-screw fixated in OLIF surgery to maintain lumbar stability, reduce interbody stress and spinal ligament tension, and provide a better fusion environment for the operative segment, but it also carries a greater risk of superior adjacent segment degeneration. How to choose the appropriate adjuvant internal fixation device in OLIF surgery and how to balance the fusion rate with the morbidity of ASD deserve our in-depth study.

## Data Availability

The data used to support the findings of this study are included within the article.
